# Effects of Cold Storage on Host *Antheraea pernyi* Egg Quality for the Egg Parasitoid *Anastatus fulloi* Sheng and Wang

**DOI:** 10.3390/insects12121057

**Published:** 2021-11-25

**Authors:** Can Zhao, Baoxin Zhang, Zixin Liu, Huiyun Zhang, Dunsong Li

**Affiliations:** 1 Guangdong Provincial Key Laboratory of High Technology for Plant Protection, Plant Protection Research Institute, Guangdong Academy of Agricultural Sciences, Guangzhou 510640, China; zhaocan@gdaas.cn (C.Z.); zhangbx@gdppri.cn (B.Z.); 2 Laboratory of Bio-Pesticide Creation and Application of Guangdong Province, Department of Entomology, College of Plant Protection, South China Agricultural University, Guangzhou 510642, China; 15581000722@163.com; 3 Institute of Tropical and Subtropical Cash Crops, Yunnan Academy of Agricultural Sciences, Baoshan 678000, China; ynkmzhy@163.com

**Keywords:** biological control, mass rearing, natural enemy, *Anastatus*, host eggs

## Abstract

**Simple Summary:**

Producing and field releasing a large number of natural enemies within a short time for pest control helps biological control programs succeed. However, host eggs are often unsynchronized with pest occurrence. Storing host eggs can help synchronize the production of parasitoids with demands of the program. *Anastatus fulloi* is an egg parasitoid wasp that has been successfully used to control the *Tessaratoma papillosa* Drury populations in south China. *Antheraea pernyi* eggs are used as factitious hosts of *Anastatus fulloi*, and cold storage of these eggs is important for large-scale rearing and successful release of *Anastatus* in the field. We studied the effects of cold storage of *A. pernyi* eggs on host egg quality and the fitness of *A. fulloi*. Cold storage reduced host egg quality and the suitability of host eggs for *A. fulloi.* To minimize losses in the large-scale rearing of *A. fulloi*, *A. pernyi* eggs should be refrigerated in a 0–3 °C or −5 °C water bath. The storage period should not exceed 6 months. The results of this study provide technical support for mass rearing of *A. fulloi* in biological control programs.

**Abstract:**

Chinese silkworm (*Antheraea pernyi*) eggs are used as factitious hosts of *Anastatus fulloi*, and refrigeration of these eggs is essential for large-scale rearing of *A. fulloi*. We studied the effects of cold storage of *A. pernyi* eggs on egg quality and the fitness of *A. fulloi* reared on the eggs. Four cold storage treatments and two cold storage periods were assessed. The 0–3 °C refrigerator treatment was unsuitable for long-term (>70 days) storage. Cold storage at −5 °C and −18 °C increased the loss rate of *A. pernyi* eggs, but there was no significant difference between the control and 0–3 °C water bath treatment. The parasitism rate of *A. fulloi* was reduced when *A. pernyi* eggs were refrigerated for 6 or 12 months. There were no obvious differences in eclosion rate and percentage of females between control and eggs subjected to 6-month storage in 0–3 °C, −5 °C, and −18 °C water bath treatments. However, the eclosion rate and percentage of females decreased sharply when the storage period was 12 months. The overall eclosion rate of *A. fulloi* was reduced at the prolonged refrigeration time. Cold storage reduced host egg quality and their fitness suitability for *A. fulloi.* To minimize losses in the large-scale rearing of *A. fulloi*, *A. pernyi* eggs should be refrigerated in a 0–3 °C or −5 °C water bath treatment, and the storage period should not exceed 6 months.

## 1. Introduction

*Anastatus* spp. are endoparasitoids of several insect orders [1,2] and are often used for the biological control of Hemiptera pests such as fruit-spotting bugs, *Amblypelta nitida* Stal and *A. lutescens lutescens* Distant [3,4,5]; citrus green stink bug, *Rhynchocoris humeralis* Thunberg [6]; litchi stink bug, *Tessaratoma papillosa* Drury [7]; and the brown marmorated stink bug, *Halyomorpha halys* Stål [8]. Mass rearing and successful field release of *Anastatus* requires a large number of host eggs [7,9]. To successfully mass rear *Anastatus* and efficiently use host material, host eggs and *Anastatus* are usually stored under cold conditions [7,9]. The development of effective storage methods that do not affect parasitoid fitness is important for successful mass rearing [10].

Several studies on *Anastatus* spp. have mentioned cold storage of host eggs and its possible effect on *Anastatus* fitness, but the most effective methods for host egg storage have not been reported [7,9,11,12]. Parasitoids reared on refrigerated eggs may have reduced fitness, because long-term cold storage of host eggs can decrease their quality [10,12,13,14,15]. A decrease in the female ratio of *Anastatus* spp. occurred when they were reared on *A. pernyi* eggs that were stored at 4 °C for 20 days [12]. A study of *Anastatus bifasciatus* Geoffroy and *Anastatus orientalis* Yang Choi showed that cold storage of host eggs did not affect their fitness [9,11]. Thus, the fitness of parasitoids can be affected by various factors.

*Anastatus fulloi* Sheng and Wang is an egg parasitoid wasp that has been successfully used to control *T. papillosa* populations in south China. *T. papillosa* is a fruit tree pest that primarily harms *Litchi chinensis* Sonn. and *Dimocarpus longan* Lour. [7,16]. Host eggs and *Anastatus* are often stored at a low temperature to meet mass rearing requirements [7]. We previously found that 10–15 °C storage of *A. fulloi* significantly decreased the wasp emergence rate after 6 months and the parasitism rate after 10 months [16]. Cold storage may not be an optimum component for mass production of *A. fulloi*. An alternative option is to store the host eggs before release. *Antheraea pernyi* eggs are usually stored at a low temperature during mass production of *A. fulloi* [7]. However, the cold storage effects on *A. pernyi* egg quality are unknown and the fitness of *A. fulloi* reared on these eggs is also not known. We studied the quality of *A. pernyi* eggs when they were stored in different cold storage conditions and the corresponding effects on the fitness of *A. fulloi*. Four cold storage treatments and two cold storage periods were evaluated.

## 2. Materials and Methods

### 2.1. Materials

Unsterilized eggs of *A. pernyi* were collected by dissecting the female moth abdomen [17,18]. *A. fulloi* were obtained from field parasitized *T. papillosa* eggs collected from a litchi orchard in Guangzhou, China. Newly emerged *A. fulloi* were fed on honey in a Perspex box (32 cm long, 25 cm wide, and 9 cm high) with a lid at 24 °C under a 16:8 h (L:D) photoperiod and 70% relative humidity (RH).

### 2.2. Cold Storage Treatments of A. pernyi Eggs

*A. pernyi* eggs were surface sterilized using a 0.1% solution of benzalkonium bromide (Bromo Geramine) for 10 min. A total of 250 g of *A. pernyi* eggs were placed into an impervious plastic bag and the opening was heat sealed. The sealed bags with fresh eggs were kept separately in a 0–3 °C water bath (the sealed plastic bag was submerged underwater, and then kept in a 0–3 °C refrigerator), 0–3 °C refrigerator (Haier, Qingdao, China; BCD−575WDBI), −5 °C freezer (Haier BC/BD−518HD), and −18 °C freezer (Haier BC/BD−518HD). After 0 (control), 6, and 12 months, 500 eggs were transferred from their storage temperature to room temperature (approximately 20–24 °C).

### 2.3. Effect of Cold Storage on the Rate of Broken A. pernyi Eggs

The 500 eggs moved to room temperature were immersed in water for 5 min. The broken eggs floated to the water surface. These eggs were scored and discarded. The percentage of broken eggs was calculated. Each treatment was repeated three times (1500 eggs total). The sinking eggs were collected for subsequent experiments.

### 2.4. Effect of Cold Storage of A. pernyi Eggs on the “Green Eggs” and Fitness of A. fulloi

Sinking eggs were surface sterilized in a 0.1% solution of benzalkonium bromide (Bromo Geramine) for 10 min, and then affixed to a paper index card (7 cm × 3.3 cm). Approximately 500 *A. pernyi* eggs were pasted on paper index cards using white glue (main chemical composition, vinyl acetate and polyvinyl alcohol). A total of 10 females and 2 males that were newly emerged (<24 h) *A. fulloi* were placed in a plastic cup (10.3 cm × 5.3 cm, H × D) and provided with *A. pernyi* eggs on the “egg card.” The plastic cups were kept at 24 °C, with a 16:8 h (L:D) photoperiod. Approximately 100 eggs were randomly sampled for the assessment of the green egg ratio, female proportion, parasitism ratio, and eclosion ratio. Green egg ratio is the proportion of green eggs among the sinking *A. pernyi* eggs. Each treatment was replicated three times. Green eggs refer to immature eggs, which were green colored. Parasitoids cannot develop normally in green eggs.

### 2.5. Data Analysis

Egg loss rate and overall eclosion rate were calculated. Egg loss rate was the proportion of broken eggs and green eggs among the *A. pernyi* eggs. Overall eclosion rate was the proportion of emerged *A. fulloi* adults in host *A. pernyi* eggs.

All of the rates were arcsine-square root transformed, and means and the standard error of untransformed data are provided in all figures. A Shapiro–Wilk test was used to check the normality of the dataset before analysis. When rates were not normally distributed, a non-parametric Kruskal–Wallis test was conducted, followed by a Mann–Whitney U test for pairwise comparisons. When the variance was shown to be equal, rates were assessed using one-way ANOVA, followed by LSD multiple comparison tests. When the variance was unequal, rates were assessed using one-way ANOVA, followed by Games–Howell multiple comparison tests. All statistical analyses were performed using SPSS 21 statistical software (IBM Inc., Armonk, NY, USA).

## 3. Results

### 3.1. Effect of Cold Storage of A. pernyi Eggs on the Broken Egg Rate, Green Egg Rate, and Egg Loss Rate

**Figure 1:**
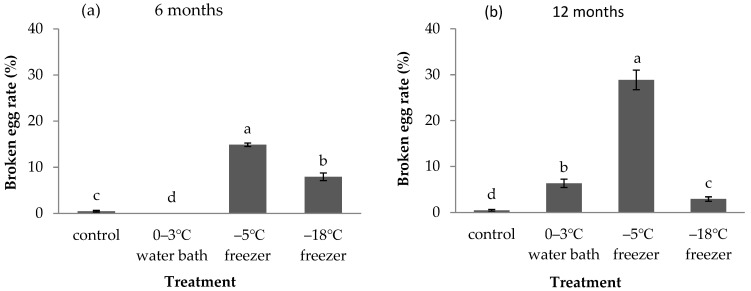
Rate of broken *A. pernyi* eggs that were stored at a low temperature for 6 months (**a**) and 12 months (**b**) (mean ± SE%). Control represents fresh eggs. Bars sharing the same letters are not significantly different (one-way ANOVA, followed by LSD multiple comparison tests, *p* < 0.05) between different treatments.

Different cold storage treatments had variable effects on the broken egg rate (Figure 1). Most *A. pernyi* eggs spoiled and had a rotten odor after 90 days storage in the 0–3 °C refrigerator. Hence, the 0–3 °C refrigerator treatment was unsuitable for long-term (>70 days) storage. As a consequence, the experiments on *A. pernyi* eggs stored in a 0–3 °C refrigerator were not continued after 70 days. The broken egg rate of *A. pernyi* eggs without storage was 0.47% (control). Significant differences occurred in the broken egg rates of *A. pernyi* eggs stored in different treatments for 6 months (F = 316.591, df = 3,8, *p* < 0.001, Figure 1a) and 12 months (F = 141.019, df = 3,8, *p* < 0.001, Figure 1b). The broken egg rate significantly increased to 14.87% when *A. pernyi* eggs were stored in a −5 °C freezer for 6 months, followed by 7.93% in a −18 °C freezer. The broken egg rate was the lowest among these three treatments when *A. pernyi* eggs were stored in a 0–3 °C water bath for 6 months. When the storage time was extended to 12 months, all of the broken egg rates significantly increased. The broken egg rate was highest when *A. pernyi* eggs were stored in a −5 °C freezer (28.87%), followed by 6.33% in a 0–3 °C water bath, and 2.93% in a −18 °C freezer. Therefore, the storage of *A. pernyi* eggs in a −5 °C freezer led to a higher broken egg rate than the other two treatments.

**Figure 2:**
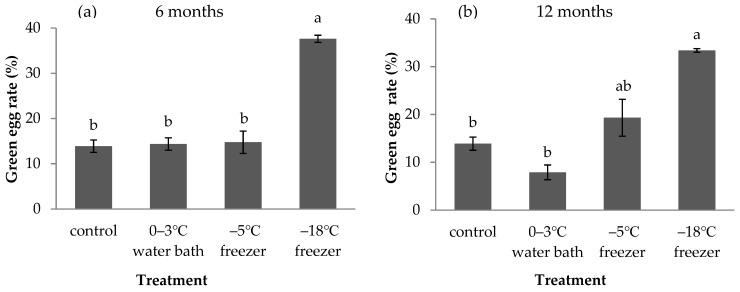
Rate of green eggs of *A. pernyi* eggs that were stored at low temperature for 6 months (**a**) and 12 months (**b**) (mean ± SE%). Control represents fresh eggs. Bars sharing the same letters are not significantly different (**a**: one-way ANOVA, followed by LSD multiple comparison tests, *p* < 0.05; **b**: one-way ANOVA, followed by Games–Howell multiple comparison tests, *p* < 0.05) between different treatments.

Different cold storage treatments had different effects on the green egg rate (Figure 2). The green egg rate of *A. pernyi* eggs without storage was 13.88% (control). A significant increase in green egg rate was observed when *A. pernyi* eggs were stored at −18 °C for 6 months (F = 34.781, df = 3,8, *p* < 0.001, Figure 2a) and 12 months (F = 20.138, df = 3,8, *p* < 0.001, Figure 2b). The green egg rate was higher when *A. pernyi* eggs were stored at −18 °C than in the 0–3 °C water bath and −5 °C freezer. There was no difference in the green egg rate of *A. pernyi* eggs among the control, 0–3 °C water bath, and the −5 °C freezer eggs. The green egg rate was higher when *A. pernyi* eggs were stored in a −18 °C freezer for 12 months than when eggs were stored in a −5 °C freezer for 12 months. However, the difference was not significant. Thus, the storage of *A. pernyi* eggs in a −18 °C freezer led to a higher green egg rate than the other two treatments.

**Figure 3:**
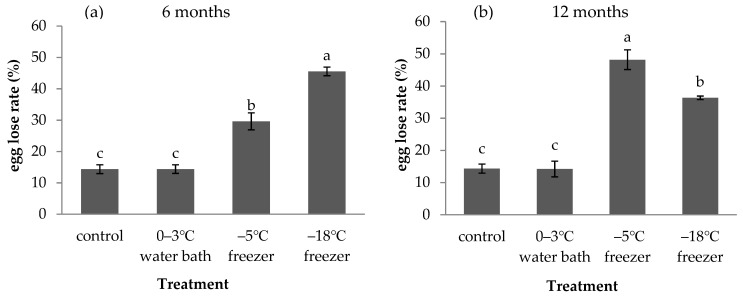
Rate of egg loss of *A. pernyi* eggs stored at low temperature for 6 months (**a**) and 12 months (**b**) (mean ± SE%). Control represents fresh eggs. Bars sharing the same letters are not significantly different (one-way ANOVA, followed by LSD multiple comparison tests, *p* < 0.05) between different treatments.

Green eggs and broken eggs were not successfully parasitized. Therefore, in this study, green eggs and broken eggs were calculated as lost eggs. The egg loss rate of *A. pernyi* eggs without storage was 14.35% (control). A significant difference was observed in the egg loss rate of *A. pernyi* eggs that were stored in different treatments for 6 months (F = 61.679, df = 3,8, *p* < 0.001, Figure 3a) and 12 months (F = 55.275, df = 3,8, *p* < 0.001, Figure 3b). When *A. pernyi* eggs were stored for 6 months, the egg loss rate was significantly increased to 45.56% in the −18 °C freezer and 29.62% in the −5 °C freezer. When *A. pernyi* eggs were stored for 12 months, the egg loss rate was also significantly increased in the −5 °C freezer and −18 °C freezer. However, there was no significant difference in the egg loss rate between the control and the 0–3 °C water bath, when *A. pernyi* eggs were stored for 6 months and 12 months.

### 3.2. Effect of Cold Storage of A. pernyi Eggs on the Fitness of A. fulloi

**Figure 4:**
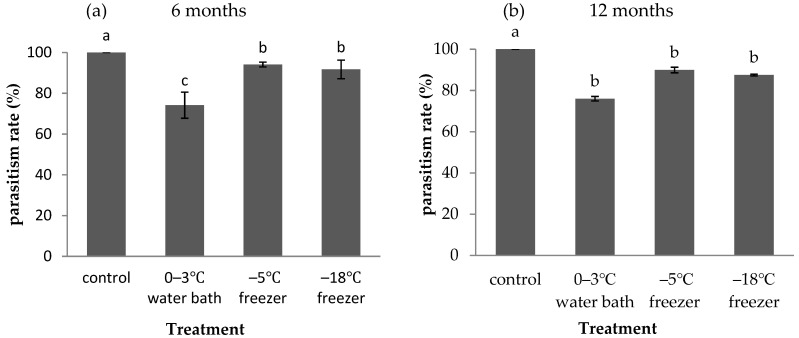
Parasitism rate of *A. fulloi* in *A. pernyi* eggs that were stored in low temperature for 6 months (**a**) and 12 months (**b**) (mean ± SE%). Control represents fresh eggs. Bars sharing the same letters are not significantly different (**a**: one-way ANOVA, followed by LSD multiple comparison tests, *p* < 0.05; **b**: non-parametric Kruskal–Wallis test, followed by Mann–Whitney U test for pairwise comparisons, *p* < 0.05) between different treatments.

Refrigeration of host *A. pernyi* eggs affected the parasitism rate of *A. fulloi* (Figure 4). The parasitism rate of *A. fulloi* in *A. pernyi* eggs without storage was 100% (control). A significant decrease in parasitism rate occurred when *A. pernyi* eggs were stored in a low temperature for 6 months (F = 15.014, df = 3,8, *p* = 0.001, Figure 4a) and 12 months (χ^2^ = 10.532, df = 3, *p* = 0.015, Figure 4b). When *A. pernyi* eggs were stored for 6 months, the parasitism rate significantly decreased to 74.1% in the 0–3 °C water bath, which was significantly lower than that in a −5 °C freezer and a −18 °C freezer. There was no significant difference in parasitism rate between the −5 °C freezer and the −18 °C freezer. When the storage extended to 12 months, the parasitism rate in the 0–3 °C water bath was lower than the parasitism rate in a −5 °C freezer and a −18 °C freezer, but there was no significant difference in the parasitism rate among the 12-month treatments.

**Figure 5:**
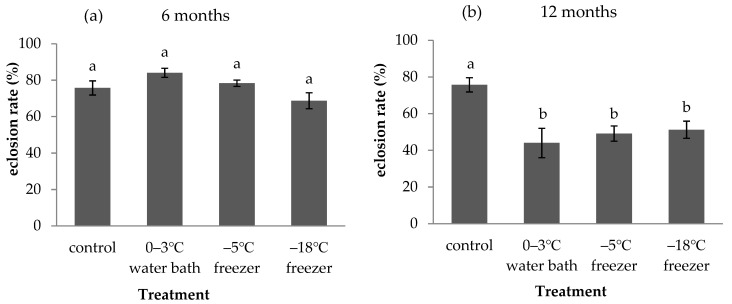
Eclosion rate of *A. fulloi* in *A. pernyi* eggs that were stored in low temperatures for 6 months (**a**) and 12 months (**b**) (mean ± SE%). Control represents fresh eggs. Bars sharing the same letters are not significantly different (one-way ANOVA, followed by LSD multiple comparison tests, *p* < 0.05) between different treatments.

Different cold storage periods of *A. pernyi* eggs had different influences on the eclosion rate of *A. fulloi* (Figure 5). The eclosion rate of *A. fulloi* in *A. pernyi* eggs without storage was 75.71% (control). When *A. pernyi* eggs were stored for 6 months in a 0–3 °C water bath, −5 °C freezer, and −18 °C freezer, there was no significant difference in eclosion rates of *A. fulloi* among these three treatments and control (F = 3.968, df = 3,8, *p* = 0.053, Figure 5a). When the storage period was extended to 12 months, the eclosion rates of *A. fulloi* significantly decreased to 44% in the 0–3 °C water bath, 49.1% in the −5 °C freezer, and 51.22% in the −18 °C freezer (F = 7.083, df = 3,8, *p* < 0.05, Figure 5b). There was no significant difference among these three treatments. 

**Figure 6:**
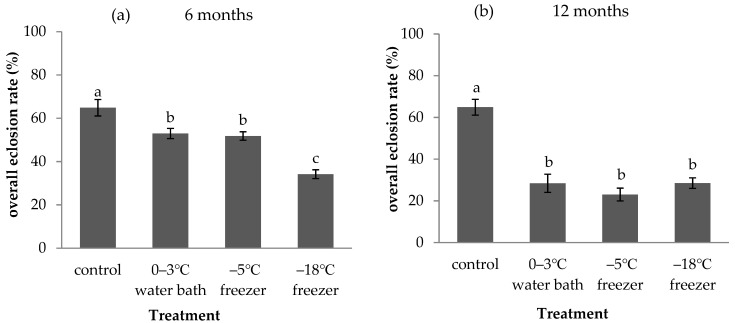
Overall eclosion rate of *A. fulloi* in *A. pernyi* eggs that were stored in low temperature for 6 months (**a**) and 12 months (**b**) (mean ± SE%). Control represents fresh eggs. Bars sharing the same letters are not significantly different (one-way ANOVA, followed by LSD multiple comparison tests, *p* < 0.05) between different treatments.

The number of parasitoids emerging from a fixed number of host eggs is important for successful mass rearing. Therefore, we compared the effect of three treatments of *A. pernyi* eggs on the overall eclosion rate of *A. fulloi*. Cold storage of host *A. pernyi* eggs had a strong effect on the overall eclosion rate of *A. fulloi* (Figure 6). The overall eclosion rate of *A. fulloi* in *A. pernyi* eggs without storage was 64.85% (control). When *A. pernyi* eggs were stored in a 0–3 °C water bath, −5 °C freezer, and −18 °C freezer for 6 months, the overall eclosion rate of *A. fulloi* significantly decreased to 52.97%, 51.8%, and 34.16%, respectively (F = 22.197, df = 3,8, *p* < 0.001, Figure 6a). There was no significant difference in the overall eclosion rate between the 0–3 °C water bath and −5 °C freezer. When *A. pernyi* eggs were stored for 12 months, the overall eclosion rate of *A. fulloi* significantly decreased to 28.4% in the 0–3 °C water bath, 23.03% in the −5 °C freezer, and 28.51% in the −18 °C freezer (F = 27.142, df = 3,8, *p* < 0.001, Figure 6b). There was no significant difference in overall eclosion rate among the three treatments. These data indicated that the host *A. pernyi* eggs should not be stored for more than 6 months.

**Figure 7:**
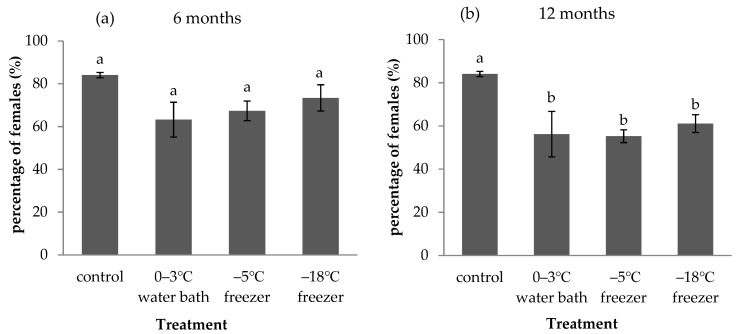
Percentage of *A. fulloi* females in *A. pernyi* eggs that were stored in low temperature for 6 months (**a**) and 12 months (**b**) (mean ± SE%). Control represents fresh eggs. Bars sharing the same letters are not significantly different (one-way ANOVA, followed by LSD multiple comparison tests, *p* < 0.05) between different treatments.

Different cold storage periods of *A. pernyi* eggs significantly affected the percentage of *A. fulloi* females (Figure 7). The percentage of *A. fulloi* females in *A. pernyi* eggs without storage was 84.06% (control). When *A. pernyi* eggs were stored in a 0–3 °C water bath, −5 °C freezer, and −18 °C freezer for 6 months, the percentage of *A. fulloi* females decreased to 63.22%, 67.34%, and 73.39%, respectively (Figure 7a). However, no difference in percentage of females was detected between the control and the three treatments (F = 2.861, df = 3,8, *p* = 0.104). When *A. pernyi* eggs were stored in a 0–3 °C water bath, −5 °C freezer, and −18 °C freezer for 12 months, the percentage of *A. fulloi* females significantly decreased to 56.2%, 55.25%, and 61.06%, respectively (F = 6.213, df = 3,8, *p* = 0.017, Figure 7b). There was no difference among the 0–3 °C water bath, −5 °C freezer, and −18 °C freezer.

## 4. Discussion

The differences in *A. pernyi* egg tolerance to low temperature may be related to their developmental maturity. *A. pernyi* eggs were not oviposited by the female moths, but collected by dissecting the eggs from the female abdomen. Some of the eggs were not fully developed and were more susceptible to harsh environments. In most of the green eggs, the egg liquid eventually dried indicating that the eggshell was poorly developed, and it could easily break and lose water. Freezing can cause expansion of the egg volume and cracking of the eggshell. This explains the greater number of broken eggs stored at −5 °C and −18 °C than those stored at 0–3 °C. Because the ice crystal nuclei grew more at −5 °C than at −18 °C [19,20], theoretically, more broken eggs would occur at −5 °C, as our data indicate. However, more green eggs were found in the eggs stored at −18 °C. This may be because most unmatured eggs were not broken at −18 °C, but suffered cryoinjury resulting in slow water loss. Accordingly, higher egg loss was calculated for the −5 °C and the −18 °C treatments, indicating that neither temperature was suitable for long-term (>6 months) storage of *A. pernyi* eggs.

Refrigerated or frozen host eggs have been successfully used for rearing many egg parasitoids [21,22,23,24]. In large-scale rearing of parasitoids, parasitism rate and eclosion rate are commonly used to estimate the suitability of host eggs [25]. Physical and chemical factors both participate in the host recognition of the parasitoids [26,27]. Storage at low temperatures can change the shape of host eggs and affect parasitoid recognition [28]. The chemical composition changes in host eggs will also reduce their acceptance by parasitoids [29]. The pH values of *Corcyra cephalonica* Stainton eggs were reduced by prolonged refrigeration at 4 °C. This change reduced the survival rate of *Trichogramma chilonis* Ishii reared on cold-stored *C. cephalonica* eggs [25]. In the present study, refrigeration reduced the suitability of *A. pernyi* eggs for *A. fulloi*. The parasitism rate significantly decreased after host egg storage in low temperature for 6 months or 12 months (Figure 4). The same results were obtained by Wu et al. [25] with eggs of rice moth stored at 4 °C for parasitism by *T. chilonis*, and by Özder [30] with eggs of *Ephestia kuehniella* Zeller stored at 0–8 °C for parasitism by *T. cacoeciae* Marchal. The decline of host egg quality and adaptability caused by storage in low temperature has also been reported in other egg parasitoids [31,32,33,34,35].

No significant difference was found in eclosion rate of *A. fulloi* when *A. pernyi* eggs were stored for 6 months (Figure 5a). Similarly, there was no significant difference in offspring emergence in *A. bifasciatus* parasitizing *H. halys* eggs stored in a −80 °C freezer for up to 24 months [9]. Broadley et al. [11] reported that field-collected spotted lanternfly eggs stored at 5 °C for 10 months did not affect the progeny production, parasitism rate, and female ratio of *A. orientalis*. Similar results were reported by Alim and Lim [22,23] with eggs of *Riptortus pedestris* Fabricius stored at 2 °C for parasitism by *Gryon japonicum* Ashmead, and *Ooencyrtus nezarae* Ishii and by Peverieri et al. [36] with eggs of *Leptoglossus occidentalis* Heidemann stored at 4 °C for parasitism by *Gryon pennsylvanicum* Ashmead. However, the eclosion rate of *A. fulloi* significantly decreased when *A. pernyi* eggs were stored for 12 months (Figure 5b). These results indicate that the nutrients and pH in host eggs remain suitable for the development of *A. fulloi* after a short period of storage. However, the nutritional composition and pH may have changed greatly after storage for 12 months, and it then became unsuitable for *A. fulloi* development. Longer cold storage may lead to deterioration of the host egg yolk and a decrease in pH, leading to reduced nutritional quality for egg parasitoids [25]. The internal changes, such as the nutritional quality of *A. pernyi* eggs after long-term storage, need further study.

Eggs stored under cold conditions will lose water content during the storage period. Water loss will lead to the changes in the physical shape of host eggs [34]. The water loss was less at −5 °C and −18 °C than in the 0–3 °C refrigerator, because the eggs were immediately frozen at −5 °C and −18 °C. Eggs stored under the 0–3 °C water bath conditions also kept well. Storage in the −5 °C, −18 °C, and 0–3 °C water baths allowed the viability of host eggs for parasitism by *A. fulloi* to extend for longer periods than eggs in the 0–3 °C refrigerator. Similar results were reported in host egg storage parasitism by *Trissolcus semistriatus* [34]. The harmful effects on the quality of host eggs and the adaptability of parasitoids appear to be decided by refrigeration conditions, refrigeration duration, and species [25].

The percentage of female wasps is an important indicator of the quality and efficacy of *Anastatus* in mass raising. The proportion of females can be affected by many factors, such as the sex ratio of the parental generation, light intensity, temperature, photoperiod, and the size and ages of host eggs. A positive relationship was reported between the size of host eggs and the proportion of females in *Anastatus* sp. [37,38]. The water and dry substance of *C. cephalonica* eggs were both reduced when the duration of refrigeration at 4 °C was prolonged [25]. The long diameter and short diameter of *C. cephalonica* eggs decreased with increased refrigeration time [39]. According to the results of these studies, we speculate that long-term storage will reduce the size of *A. pernyi* eggs, resulting in a decrease in the proportion of *A. fulloi* females. This study showed no significant difference in the proportion of *A. fulloi* females between fresh eggs and eggs that were stored for 6 months under three treatments (Figure 7). However, the proportion of *A. fulloi* females significantly decreased when the storage period was extended to 12 months. Zhou et al. [12] also reported a decrease in the proportion of *Anastatus* females when *A. pernyi* eggs were refrigerated at 4 °C for 20 days. However, there was no difference in the proportion of *T. chilonis* females when *C. cephalonica* eggs were stored at 4 °C for 0–45 days [25].

## 5. Conclusions

Cold storage at −5 °C and −18 °C resulted in an increased loss rate of *A. pernyi* eggs used for the mass rearing of *A. fulloi,* but no significant differences were observed among the fresh eggs and 0–3 °C water bath treatment. Cold storage in all three treatments led to reduced fitness of *A. pernyi* eggs for large-scale rearing of *A. fulloi.* To minimize losses in the large-scale rearing of *A. fulloi*, *A. pernyi* eggs should be refrigerated in a 0–3 °C or −5 °C water bath, and the storage period should not exceed 6 months. When the storage period is extended to 12 months, both the overall eclosion rate and female rate are significantly decreased and these would reduce *A. fulloi* production.
